# 337 Joy of Minimizing Overuse (JOMO): Outcomes of Antimicrobial Stewardship Interventions on Antibiotic Use for Diabetic Foot Infections

**DOI:** 10.1017/ash.2026.10739

**Published:** 2026-06-23

**Authors:** Diana Vilar-Compte, Pamela Graciadiego-Fossas, Ana Cecilia Carbajal-Cesar, Itzel Stella P豥z-Campos, Cyntia Ibanes Guti豲ez, Juan Pablo Ramirez-Hinojosa, Elida Alonso-Tr୳ito, Arturo Galindo-Fraga

**Affiliations:** 1 Instituto Nacional De Cancerologia; 2 Hospital Infantil de México; 3 Instituto Nacional de Pediatría; 4 Hospital General Dr. Manuel Gea GonzLez

## Abstract

**Background:** Measles cases increased significantly in the Americas in 2025; the region lost its measles-free status due to the return of endemic transmission in Canada, Mexico, and the U.S., primarily driven by low vaccination rates among unvaccinated or under-vaccinated populations. In August 2025, Mexico City experienced a surge in measles cases, prompting an urgent call to boost immunization coverage among targeted groups through mass vaccination of children, adolescents, healthcare workers (HCWs), and adults under 50. We report post-vaccination mumps cases among HCWs from six hospitals. **Methods:** A retrospective study of individuals vaccinated with the Serum Institute of India measles, mumps, and rubella (MMR) vaccine, which contains the Leningrad-Zagreb (L-Z) strain, was conducted at six national referral tertiary care hospitals affiliated with the Mexican Ministry of Health. We included all individuals under 50 years who, 10-30 days after vaccination, developed salivary gland swelling with or without malaise, headache, lymphadenopathy, or fever. We performed a descriptive analysis and calculated the incidence rate of vaccine-related mumps per 1000 doses of the MMR vaccine. **Results:** Between 08/21/2025 and 11/30/2025, 10,590 HCWs received the Serum Institute of India MMR vaccine; 27 (2.5 X MMR 1000 vaccines) developed parotid swelling (7 bilateral, 20 unilateral), with concomitant fever in 7 (25.9%); lymphadenopathy, 10 (37%); pharyngitis, 11 (40.7%); arthralgias, 8 (29.6%); and headache, 11 (40.7%). One person developed a rash and malaise. The median time from vaccination to mumps was 16.8 days. No cases of meningitis or other complications were reported. The average duration of symptoms was 8.2 days. No secondary mumps cases were detected. Discussion: Post-vaccination mumps and aseptic meningitis after MMR vaccination are rare and infrequently reported, though prevalence varies by strain. Despite similar efficacy among MMR vaccines, the Serum Institute of India vaccine includes the L-Z strain, which carries a higher risk of mumps (approximately 4.4%) than the Jeryl-Lynn strain used in the MMR-II® (0.5-1.6%), which is primarily used in high-income countries. In our cohort, the prevalence of post-vaccine mumps with the Serum Institute of India MMR, a lower-cost vaccine, was low and consistent with other reports. No cases of aseptic meningitis were reported. With the rising number of measles cases worldwide and the need to increase vaccination coverage against measles in under-vaccinated populations, reinforcing the similar efficacy and safety of different vaccines is crucial. All HCWs must be protected.

